# A Three-dimensional Printed Low-cost Anterior Shoulder Dislocation Model for Ultrasound-guided Injection Training

**DOI:** 10.7759/cureus.3536

**Published:** 2018-11-02

**Authors:** Zachary Risler, Mark A Magee, Jacob M Mazza, Kelly Goodsell, Arthur K Au, Resa E Lewiss, Robert S Pugliese, Bon Ku

**Affiliations:** 1 Emergency Medicine, Thomas Jefferson University, Philadelphia, USA

**Keywords:** medical simulation, 3d printing, anterior shoulder dislocation, emergency medicine

## Abstract

Anterior shoulder dislocations are the most common, large joint dislocations that present to the emergency department (ED). Numerous studies support the use of intraarticular local anesthetic injections for the safe, effective, and time-saving reduction of these dislocations. Simulation training is an alternative and effective method for training compared to bedside learning. There are no commercially available ultrasound-compatible shoulder dislocation models. We utilized a three-dimensional (3D) printer to print a model that allows the visualization of the ultrasound anatomy (sonoanatomy) of an anterior shoulder dislocation.

We utilized an open-source file of a shoulder, available from embodi3D*® *(Bellevue, WA, US). After approximating the relative orientation of the humerus to the glenoid fossa in an anterior dislocation, the humerus and scapula model was printed with an Ultimaker-2 Extended+ 3D® (Ultimaker, Cambridge, MA, US) printer using polylactic acid filaments. A 3D model of the external shoulder anatomy of a live human model was then created using Structure Sensor®(Occipital, San Francisco, CA, US), a 3D scanner. We aligned the printed dislocation model of the humerus and scapula within the resultant external shoulder mold. A pourable ballistics gel solution was used to create the final shoulder phantom.

The use of simulation in medicine is widespread and growing, given the restrictions on work hours and a renewed focus on patient safety. The adage of “see one, do one, teach one” is being replaced by deliberate practice. Simulation allows such training to occur in a safe teaching environment. The ballistic gel and polylactic acid structure effectively reproduced the sonoanatomy of an anterior shoulder dislocation. The 3D printed model was effective for practicing an in-plane ultrasound-guided intraarticular joint injection.

3D printing is effective in producing a low-cost, ultrasound-capable model simulating an anterior shoulder dislocation. Future research will determine whether provider confidence and the use of intraarticular anesthesia for the management of shoulder dislocations will improve after utilizing this model.

## Introduction

In 2011, there were over 175,000 emergency department (ED) visits nationally for shoulder dislocations, accounting for an incidence of 23.9 per 100,000 person-years [1-4]. The majority of these patients are definitively managed in the emergency setting in up to 92% of cases [5]. As such, the emergency physician must commonly diagnose and manage this presentation.

Studies have shown that intraarticular injections of local anesthetic are safe, effective, time-saving, and cost-saving as compared to intravenous sedation for the reduction of dislocated shoulders [6-10]. Ultrasound-guided intra-articular injection is effective yet not routinely used [11].

The use of simulation models has long been accepted as a method for training physicians, with particular success for learning procedures [12]. Models currently exist to train practitioners in ultrasound-guided procedures, such as vascular access, nerve blocks, paracentesis, and thoracentesis. Currently, there are no commercially available ultrasound-compatible models of a shoulder dislocation, allowing for intraarticular aspiration and injection. Furthermore, when available, commercial models can be expensive, about $400-$550 for simple vascular models and close to $4000 for lumbar puncture models [13].

Three-dimensional (3D) printing is an efficient and affordable way to create simulation models. 3D models for procedure learning and competency assessment are available to model anatomy, practice procedures, and plan surgical interventions [14-16]. As mentioned, there are no ultrasound-compatible shoulder dislocation models. The goal of our educational innovation was to build a novel model to teach the ultrasound identification of anterior shoulder dislocation.

This report was previously presented at American Institute of Ultrasound in Medicine Annual Convention in 2018 (Oral abstract: Mazza J, Risler Z, Magee M, Goodsell K, Pugliese R, Ku B. A Novel 3-Dimensional Model for Teaching Shoulder Dislocation Identiﬁcation and Intra-Articular Injections. American Institute of Ultrasound in Medicine 2018 Annual Convention; March 26, 2018).

## Technical report

**Figure 1:**
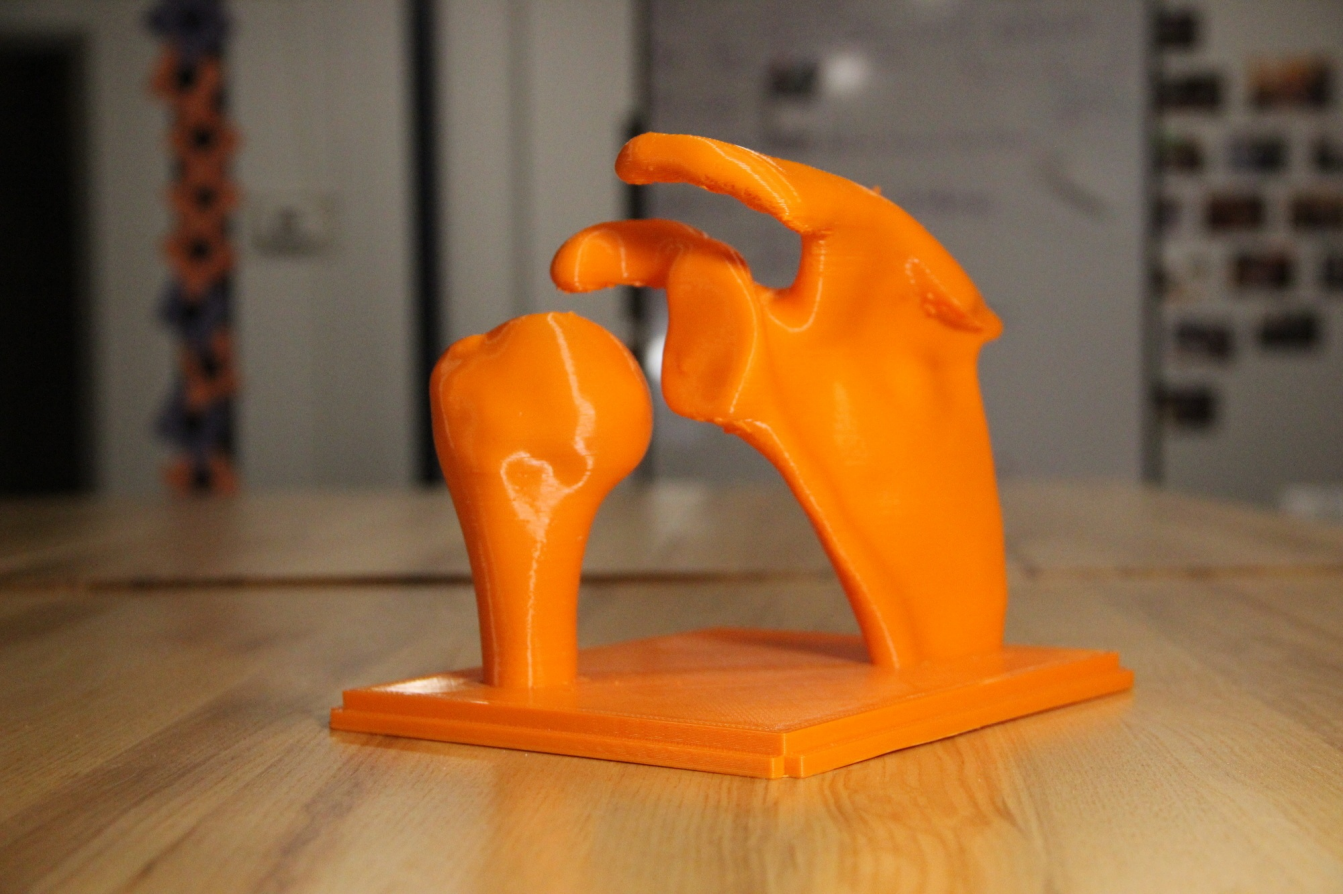
3D printed shoulder joint from the back

**Figure 2:**
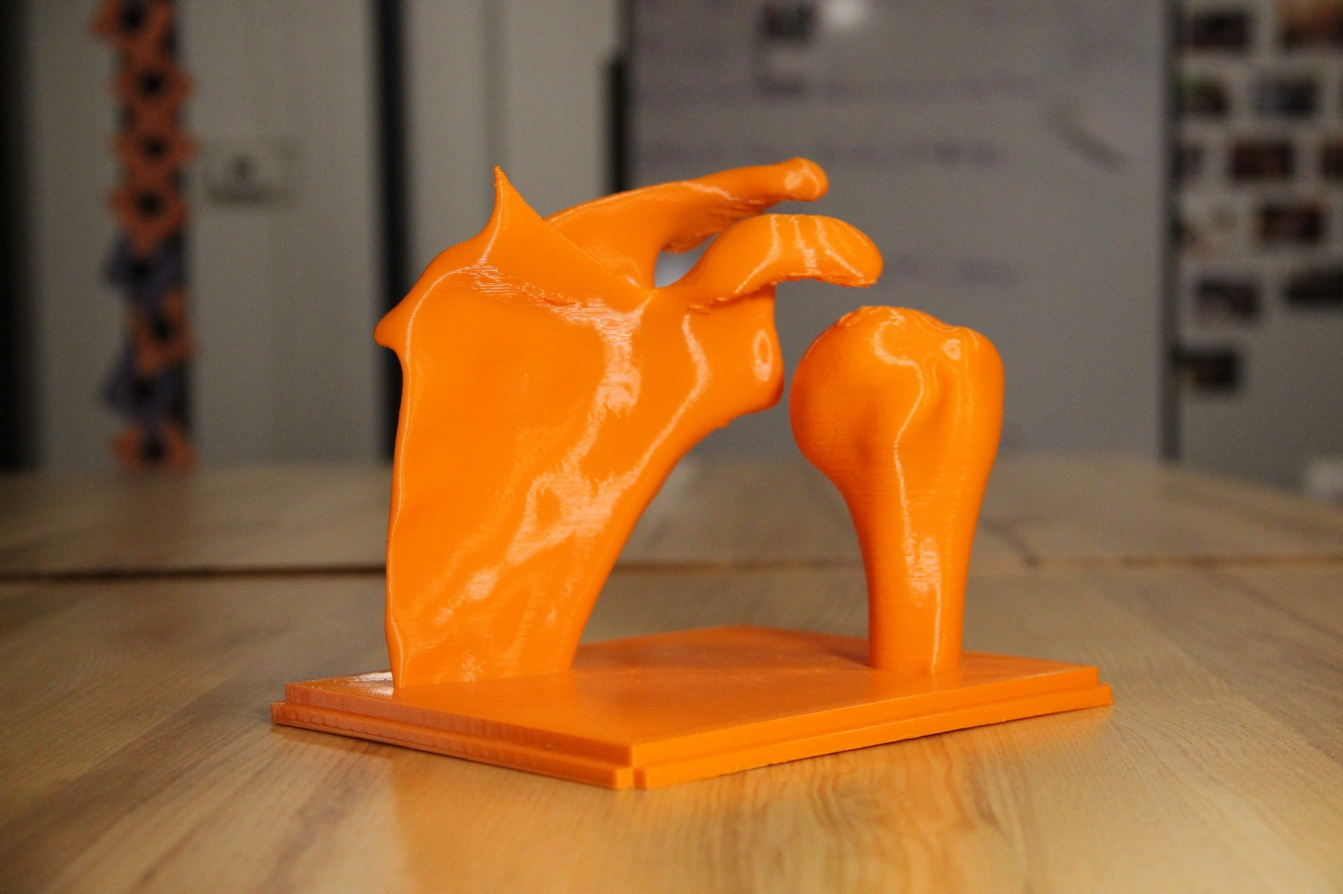
3D printed shoulder joint from the front

**Figure 3:**
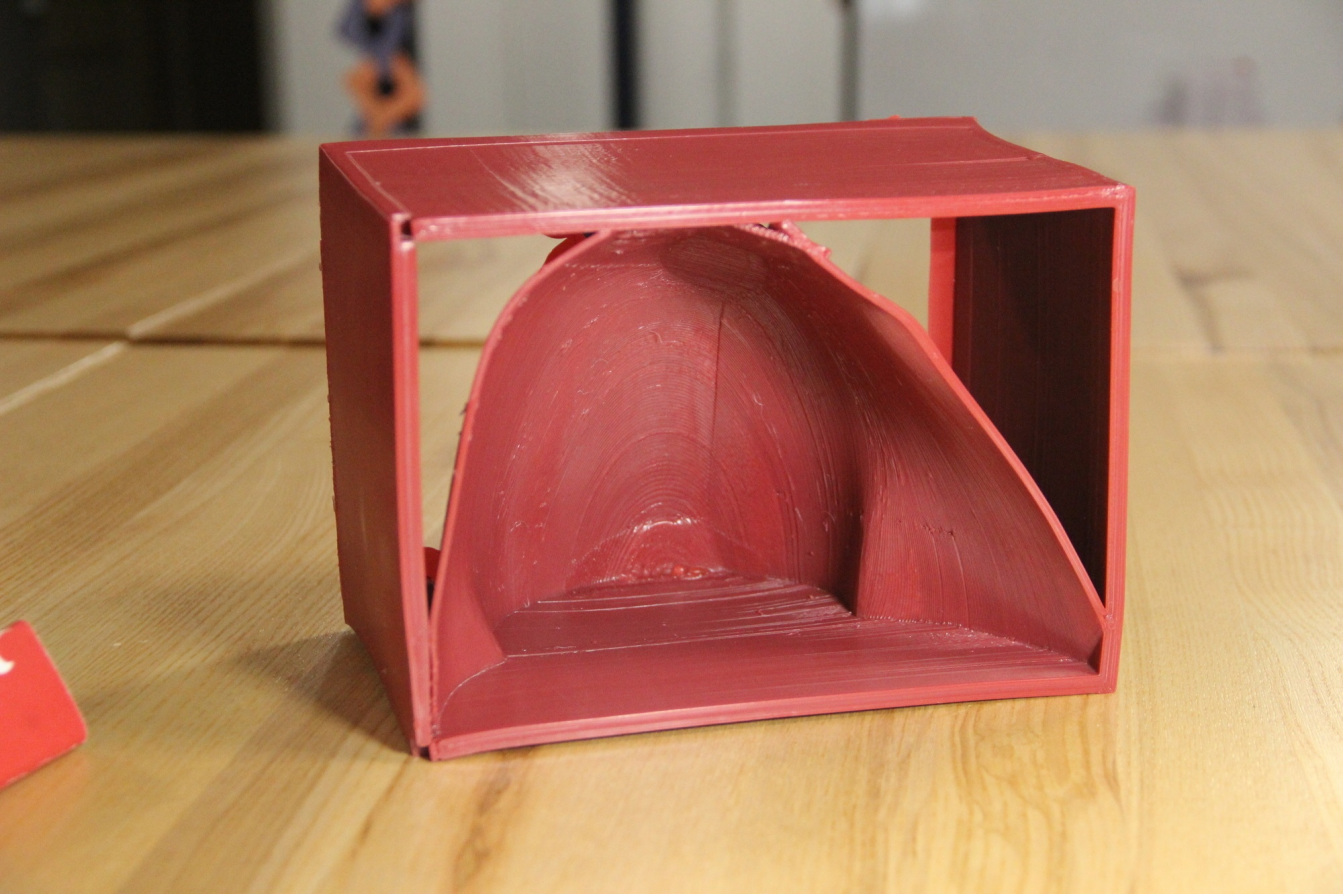
3D printed shoulder mold

**Figure 4:**
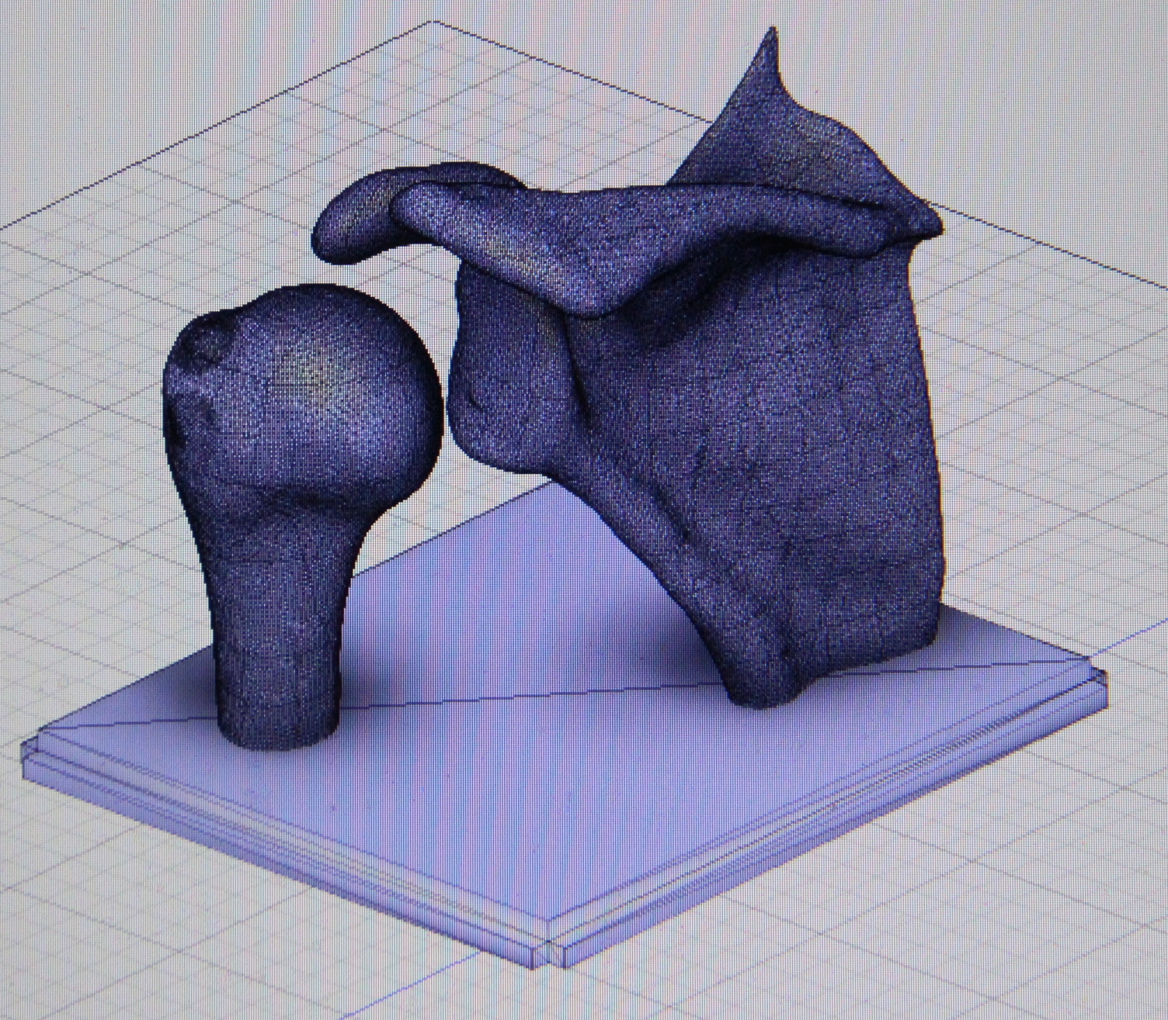
3D computer rendering of shoulder joint

The life-size shoulder model was constructed using two parts: the joint model (Figures 1-2) and the shoulder mold (Figure 3). The shoulder joint model was made utilizing open-source files available from embodi3D® (Bellevue, WA, US) [17]. Models of a human left scapula and proximal humerus were imported into Autodesk Fusion 360® (Autodesk, San Rafael, CA, US), a software program that allows for the manipulation of the imported model. The two bone models were manipulated in the software to represent the correct spatial positioning of a human anterior shoulder dislocation (Figure 4). In addition to the correct spacing, a flat, rectangular base was modeled in Fusion 360 and added to the bone model to serve as a base support. Once the combined humerus-scapula model was created in Fusion 360, the file was exported as a .STL file and imported into Ultimaker Cura® (Ultimaker, Cambridge, MA, US), an open-source 3D printer slicing application, for preprinting processing. In Cura, appropriate printing parameters were set for the Ultimaker 2+ Extended® 3D printer, and a .gcode file was generated to print the shoulder bone model. A co-author (JM) independently learned the above software utilizing free online guides and was the lead in the production of the shoulder model.

**Figure 5:**
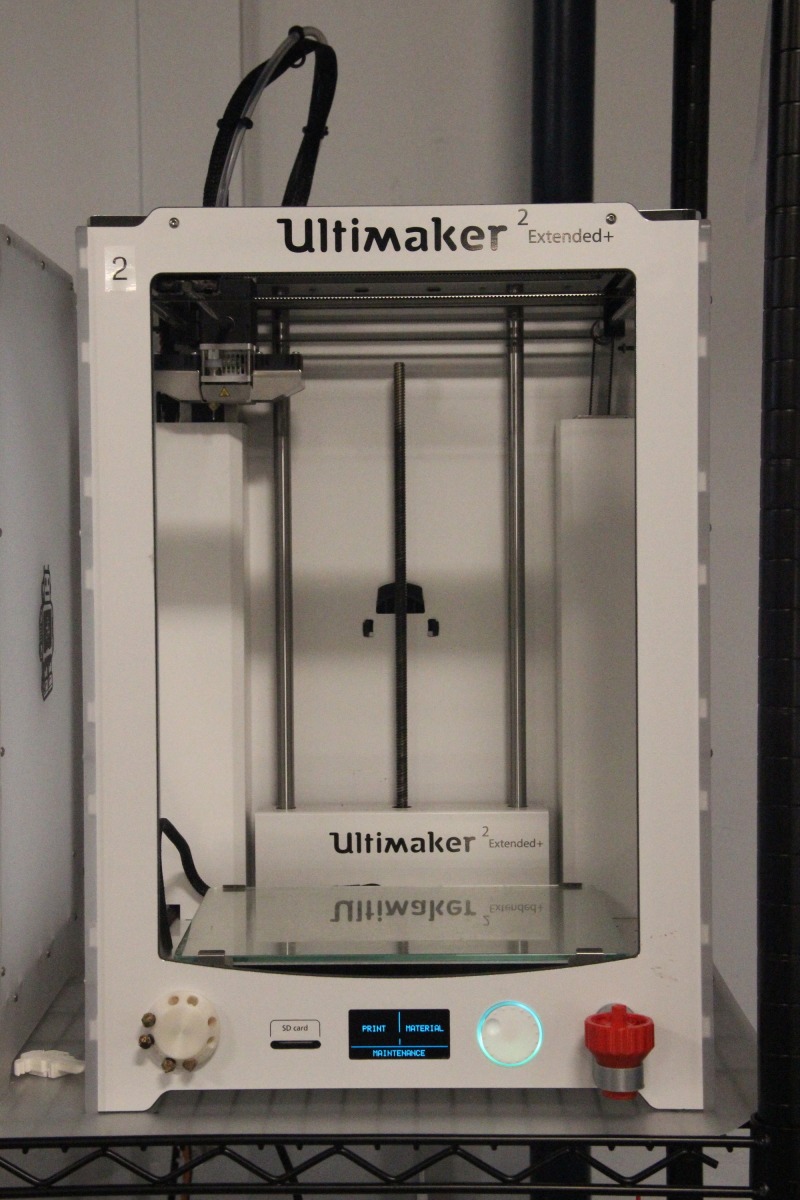
Ultimaker 2 extended 3D printer Ultimaker 2 extended 3D printer: Ultimaker, Cambridge, MA, US

Using an Apple iPad Pro® (Apple, Cupertino, CA, US) equipped with a Structure 3D® scanner (Occipital, San Francisco, CA, US), a 3D scan of a male, left shoulder was obtained to create the shoulder mold. The scanned image file was processed in Autodesk Meshmixer® (Autodesk, San Rafael, CA, US) for the proper sizing and orientation of the model to be printed. Once complete, a .STL file of the shoulder mold was imported into Cura for preprinting processing. A .gcode was created for the shoulder mold to be printed on the Ultimaker 2+ Extended® (Figure 5).

Using the Ultimaker 2+ Extended, both the shoulder joint and shoulder mold models were printed using polylactic acid (PLA) to human scale. Printer settings were optimized to reduce cost and time, including using a 0.8mm printer nozzle and adjusting the infill density of the prints to 20%. No post-printing processing was required for either the joint or mold models.

**Figure 6:**
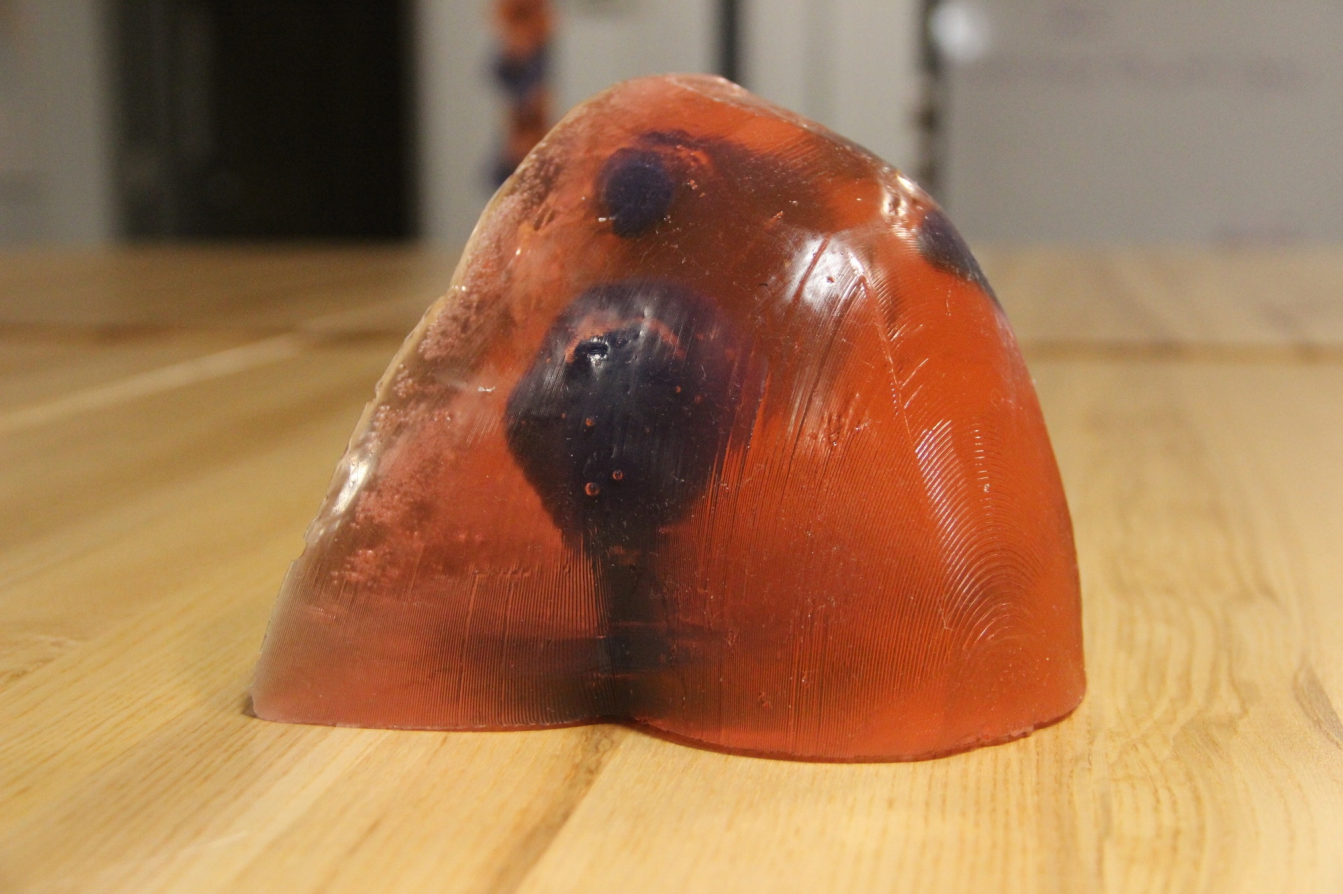
Final model from front

**Figure 7:**
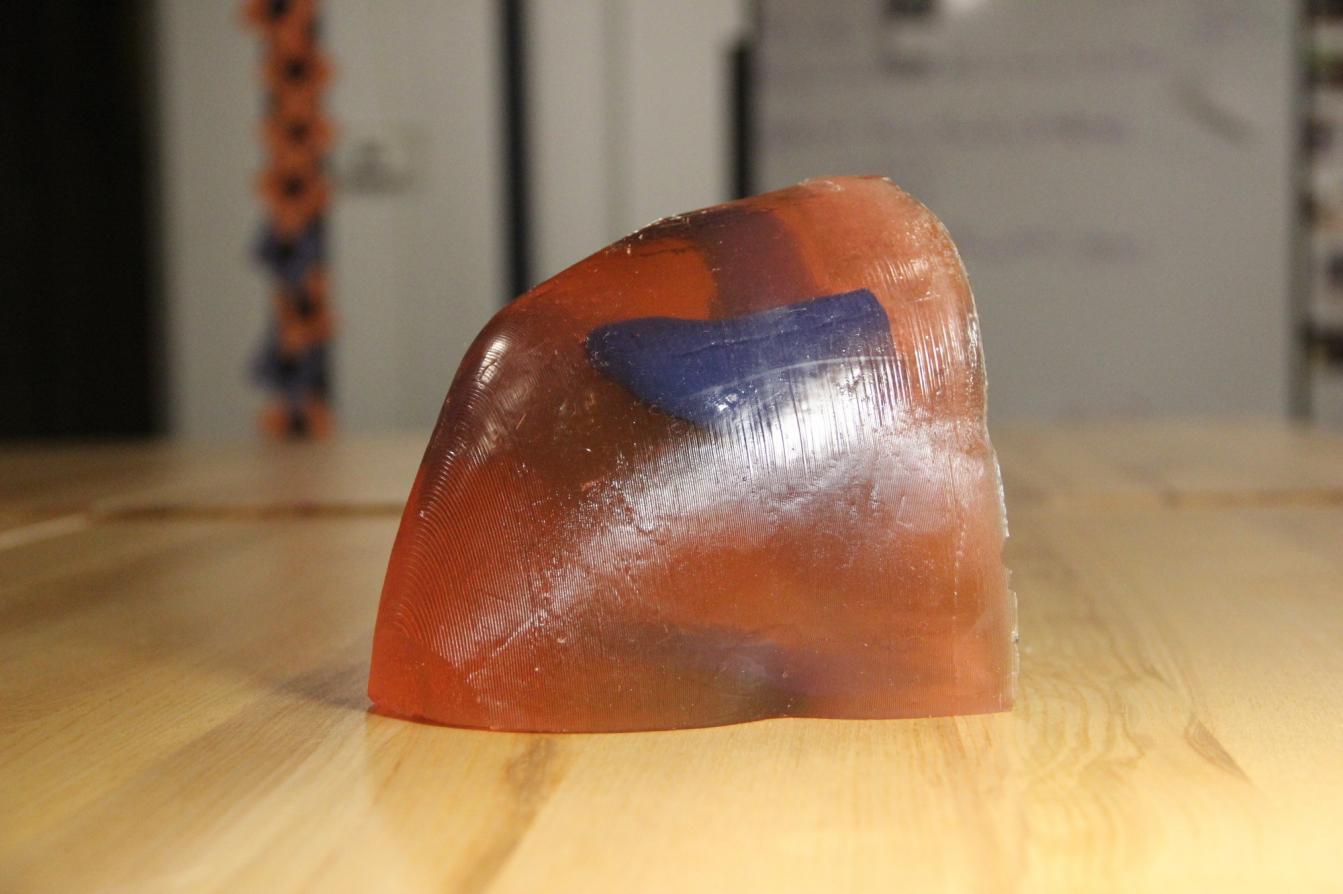
Final model from back

The next step in the creation of the final shoulder model was casting to replicate the soft tissues of the shoulder. To cast the shoulder phantom, two pounds of medical-grade ballistics gel (www.humimic.com) resembling average human soft tissue was cut into 1 cm x 1 cm cubes and placed in a lab-grade glass beaker. The gel was heated in a standard convection oven for six hours at 135C. Two coats of mold release spray were applied to the shoulder mold, five minutes apart from one another and five minutes before pouring the mold. Once the gel was thoroughly melted and there were no visible bubbles within the bulk of the liquid gel, the gel was poured into the 3D printed shoulder mold at a slow, steady pace so as to not introduce bubbles. Immediately after pouring, the 3D printed shoulder joint model was slowly submerged into the liquid gel at the proper position. In order to keep the bones in the proper position throughout cooling, two paint stirrers were positioned over the base of the bones and shoulder mold, with a metal weight positioned on top of the stirrers so that the bones could not rise up in the liquid gel. The shoulder mold with gel and bones sat to cool for 12 hours. Once cooled, the gel phantom was carefully pulled out of the shoulder mold, ready to use (Figures 6-7). The total cost of the model including the ballistic gel was approximately $60.

**Figure 8:**
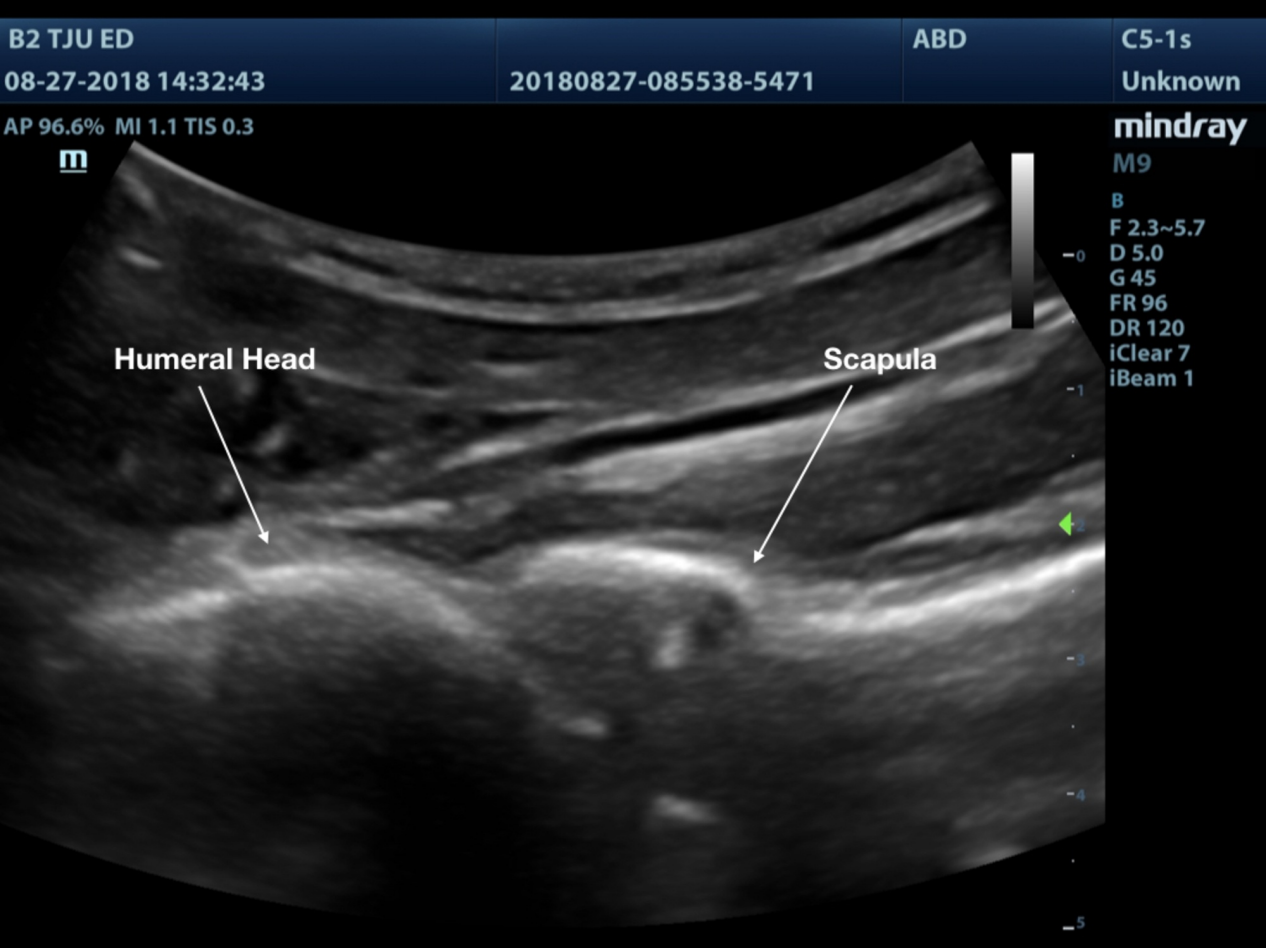
Shoulder joint with normal anatomic alignment

**Figure 9:**
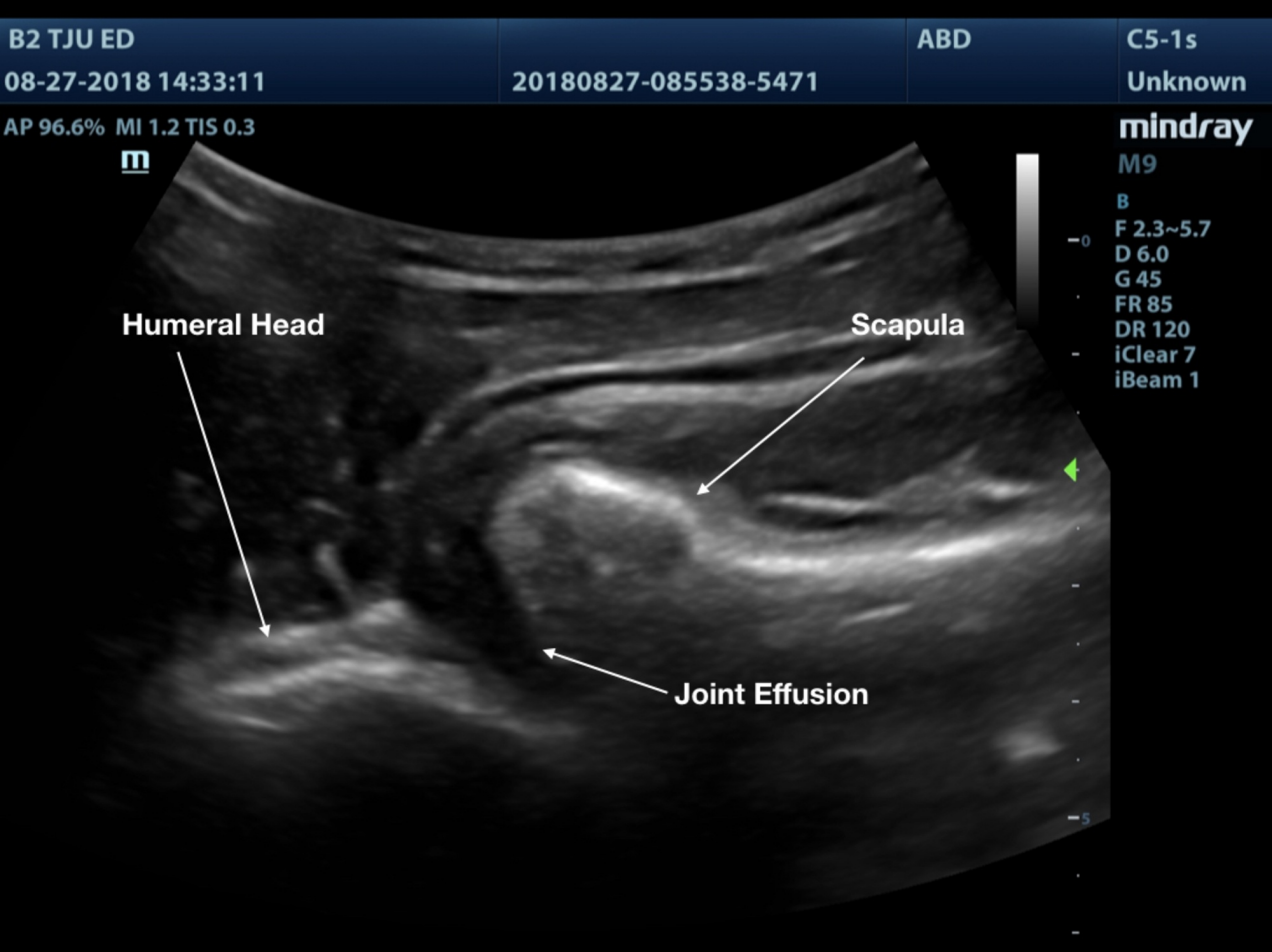
Shoulder joint with anterior dislocation

**Figure 10:**
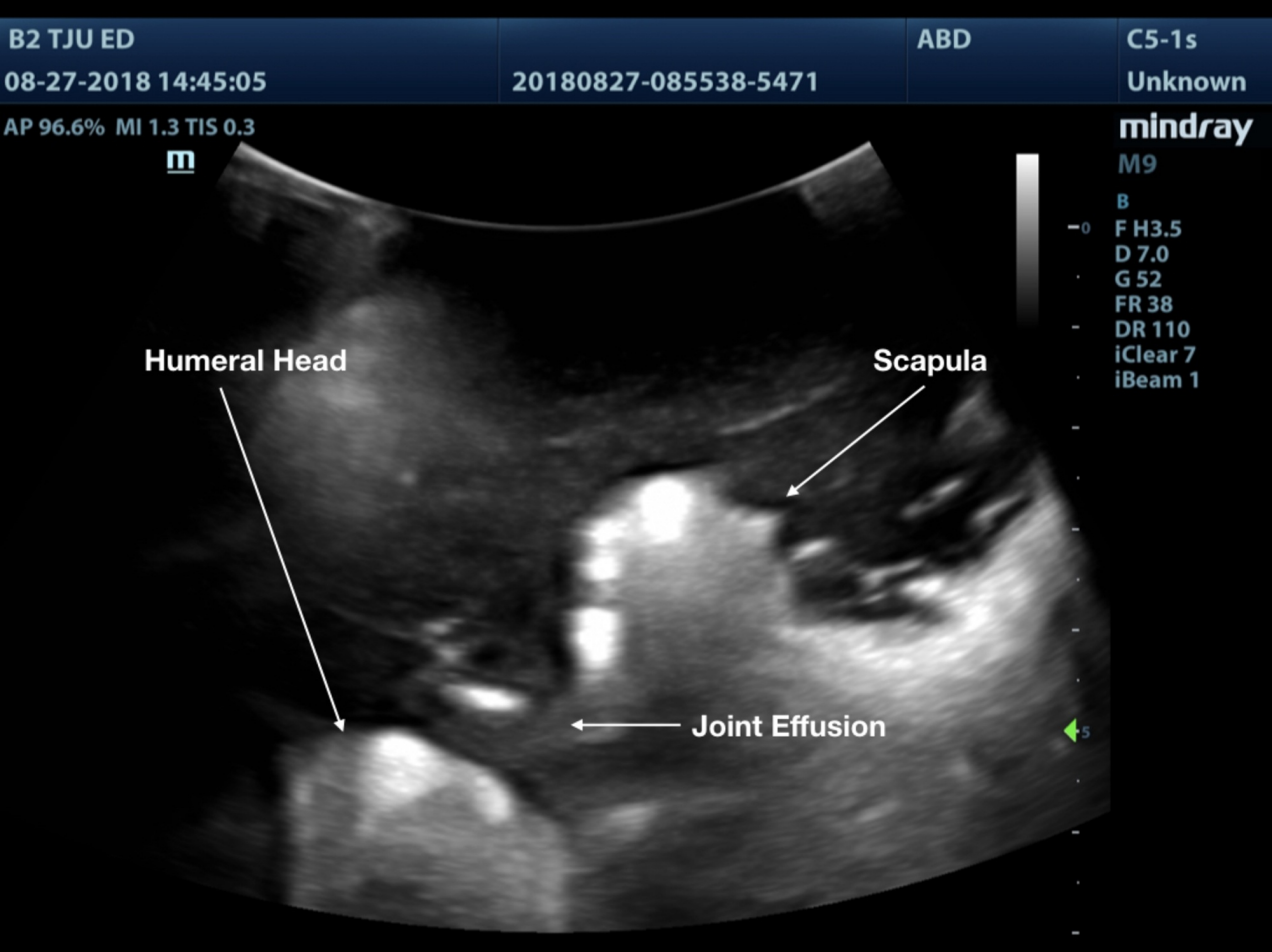
3D printed shoulder joint with anterior dislocation

**Figure 11:**
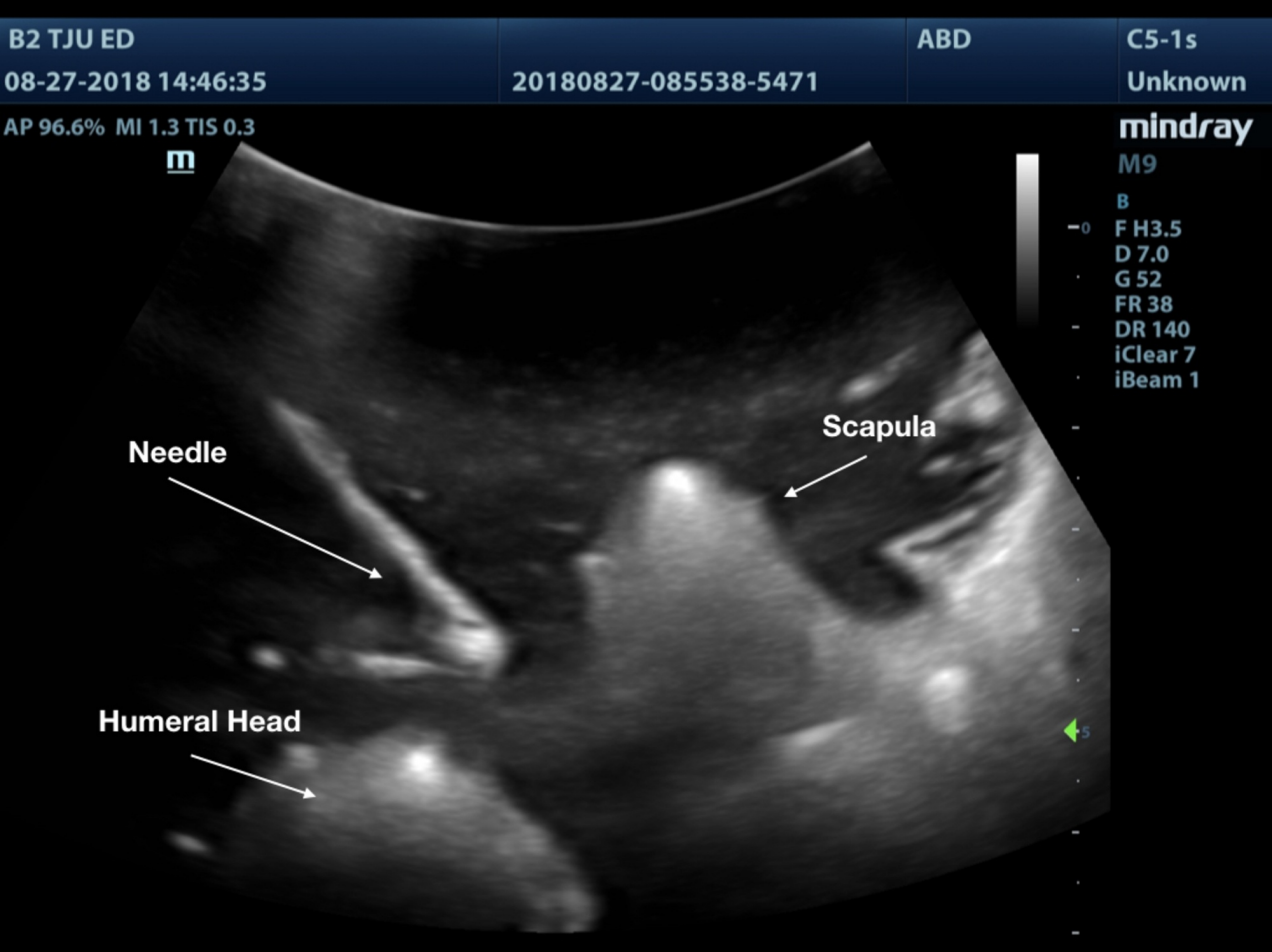
3D printed anterior dislocated shoulder showing needle within the joint space

A 3D printed shoulder model was successfully printed using the technique outlined above. The model produced images that are comparable to a true anatomical scan (Figures 8-10). The 3D printed model in the ballistic gel was effective in reproducing the finding of an anterior shoulder dislocation, as expected with ultrasound imaging. The model also allowed for the visualization of the needle within the joint space (Figure 11).

## Discussion

The use of simulation in medicine is widespread and growing, given the restrictions on work hours and a renewed focus on patient safety. The adage of “see one, do one, teach one” is being replaced by deliberate practice [18]. Simulation allows such training to occur in a safe teaching environment.

Simulation has been studied in many medical fields, particularly surgical subspecialties in which technical skills are paramount. A study by Nesbitt and colleagues compared fourth-year medical students and senior surgical residents in performing a coronary anastomosis. The fourth-year medical students were instructed through deliberate simulation practice and one-on-one training while the surgical residents were given a single lesson. The study found that there was no significant difference between overall score and time to completion. The authors concluded that deliberate practice allowed for proficiency in task completion [19].

Another study by Zendejas and colleagues compared residents performance of total extraperitoneal hernia repair using an apprenticeship model, widely used today in medicine, versus a mastery learning practice, which involved online modules and simulation. The study found that the simulation-based group had decreased operative time, improved trainee performance, decreased intraoperative and postoperative complications, and reduced overnight stays post procedure [20].

While simulation has become more complex, similarly simulators have become more complex. As commercial simulators have become more advanced and expensive, a need for more accessible simulators has developed. Our simulator cost about $60 for supplies, not including the price of the printer. While it took some time to manipulate the file to be printed, this will become much easier with additional open access sources that are being created. We believe that 3D printing will allow for customizable and inexpensive simulation. Due to the similarity of ultrasound imaging of the PLA and bone, 3D printing is a great resource in ultrasound simulation.

There were several limitations to our model identified, the most significant of which was the need for a 3D printer. While purchasing a printer requires a significant investment, the cost savings over time can be great as the need to purchase commercial models is reduced. Furthermore, additional models can be made with open-source files readily available on the Internet. Our model did not have a reservoir to aspirate fluid, as is ideal in the actual procedure. We plan to add this feature to the next generation of the model. Lastly, production of the model was time-consuming but future attempts will be faster given experiences gained in previous use. However, this needs to be weighed against the cost of other models and the customizability of the model.

Next steps

The model provided appropriate images to demonstrate an anterior shoulder dislocation. Next steps would be to improve imaging and add a reservoir to allow for aspiration and injection. Next, the model could be used in a curriculum to assess if it improves comfort with the procedure, procedure success rate, and, ultimately, improve pain scores and decrease the use of sedation for shoulder dislocations within the emergency department.

## Conclusions

The use of 3D printing is effective in producing a low-fidelity, ultrasound-capable model simulating an anterior shoulder dislocation. Future research will determine whether provider confidence will improve after utilizing this model and whether provider interactions with the model increases the frequency of use of intraarticular anesthesia for the management of shoulder dislocations in our department.
